# The Ohio State Veterinary Medical Association

**Published:** 1896-03

**Authors:** 


### THE OHIO STATE VETERINARY MEDICAL ASSOCIATION.

THE Secretary, Dr. W. H. Gribble, Washington C. H., on February 13, 1896, addressed the following circular to veterinarians in his State:

"At the last annual meeting it was voted to meet again during the coming summer in Columbus. Since that time it has been announced that the United States Association would meet in Buffalo, N. Y., on September 1st, 2d, and 3d, and your Secretary has received several letters asking if our meeting could not be postponed to the same place and time as the U. S. V. M. A. Every member of Ohio's Association is requested to write us giving his views in the matter. The Committee on Law, composed of Drs. W. H. Gribble, W. R. Howe, and J. D. Fair, on February 11, 1896, had D. R. Death arrested in Dayton, O., charged with illegally practising veterinary medicine and surgery. This is the first arrest under the law governing the practice of veterinary medicine in this State, and the defendant was fined ten dollars and costs, the minimum fine under the law."

